# Introduction to “Intramolecular Hydrogen Bonding 2018”

**DOI:** 10.3390/molecules24162858

**Published:** 2019-08-07

**Authors:** Goar Sánchez

**Affiliations:** Irish Centre for High-End Computing (ICHEC), Grand Canal Quay, Dublin 2, Ireland; goar.sanchez@ichec.ie

Non-covalent interactions have attracted the scientific attention during last decades as observed by the numerous studies in the literature [1,2,3,4], however, those are still hot topics. There is wide variety and rich ecosystem of non-covalent interactions: Hydrogen bonds [5], halogen bonds [6], chalcogen bonds [7,8,9,10], pnicogen bonds [11,12,13], tetrel bonds [14,15,16] and more recently, regium bonds [17].

Doubtless, the most prominent, and at the same time studied, interaction is the hydrogen bond (HB) [5]. Not only for the availability of the atoms involved within the interactions but also for the versatility and presence of those HB across all fields, from protein shaping, DNA and RNA assembling, proton transfer processes, crystallisation, and without any doubt, in synthesis. For example, HBs are also of utmost importance in water nucleation and responsible of the variety of ice structures. In fact, Peng et al. studied the vibrations and spectra of ice X and XIV using Density Functional Theory (DFT) methods [18,19]. Another example of the importance of HBs are their influence on the solubility of drugs during the synthetic process. This is particularly important since impurities can be introduced during the synthesis which can have a huge impact on the crystallisation process. Shen et al. measured and modelled the solubility of Metoprolol succinate in different organic solvents involving HBs [20]. Also, HBs play a crucial role in supramolecular chemistry and molecular recognition. Martinez-Felipe et al. combined Fourier transform infrared spectroscopy and DFT calculations to study the molecular recognition using HB in light-responsive materials for optical and light-controlled delivery applications [21].

One particular and very interesting case of HBs occurs when the hydrogen acceptor and donor are within the same molecular unit, i.e., intramolecular hydrogen bond (IMHB). Intramolecular hydrogen bonds are known to play an important role on the structure and biological properties of compounds in medicinal chemistry [22,23]. As for example, IMBH formation is crucial in the process of diffusion of cyclic peptides through cell membranes [24]. However, IMHBs, as other intramolecular interactions, particularly difficult to analyse and measure, and thus, computational calculations in combination with NMR measures can provide reliable data to understand the nature and strength of such interactions [25].

As mentioned, HBs (both inter and intramolecular) are present in proton transfer processes [26,27], and can determine reaction rates and lower reaction barriers. Tschumper et al. studied the importance of IMHBs in the cis/trans conformation equilibrium for aziridine, phosphirane and thiirane analogs of 1,2-dialkyl-2,3-epoxycyclopentanol, where not only typical IMHBs can be found (O–H···O) but also another HB acceptors, such as S or P, can be found [28].

Another interesting feature found within non-covalent interactions happens when those interactions are conjugated with the carbon backbone and the interaction is affected by the presence of the resonant structures. Particularly, resonance assisted hydrogen bonds (RAHB) are one of the most successful and interesting structural concepts, since its definition by Gilli, Bellucci, Ferretti and Bertolasi in 1989 [29]. Several studies have been devoted to the study of RAHBs, both for inter [30] and intramolecular RAHB [31,32,33]. In fact, Martínez-Cifuentes et al. have studied the strength of intramolecular resonance assisted hydrogen bonding in o-carbonyl hydroquinones [25].

Finally, the interaction between HBs and other type of non-covalent interactions opens a new research line in which one of the interactions (whether HB or IMHB) can be influenced by the presence of another type of interactions or the other way around. This interplay can be co-operative, in which one of the interactions reinforces or enhances the other [34,35,36,37,38]. In the present issue, Alkorta et al. studied how the presence and absence of IMHB can affect the tetrel bond in a series of silyl and germanium derivatives [39].
